# Culture Time Needed to Scale up Infrapatellar Fat Pad Derived Stem Cells for Cartilage Regeneration: A Systematic Review

**DOI:** 10.3390/bioengineering7030069

**Published:** 2020-07-04

**Authors:** Sam L. Francis, Angela Yao, Peter F. M. Choong

**Affiliations:** 1 Department of Surgery, The University of Melbourne, Melbourne, VIC 3065, Australia; pfchoong@gmail.com; 2 Department of Orthopaedics, St Vincent’s Hospital, Melbourne, VIC 3056, Australia; angela.yao.inbox@gmail.com; 3 Biofab 3D, Aikenhead Centre for Medical Discovery, Melbourne, VIC 3065, Australia

**Keywords:** stromal vascular fraction, stem cell, adipose-derived stem cell, infrapatellar fat pad, knee, arthroscopy, arthrotomy, tissue engineering

## Abstract

Adipose tissue is a rich source of stem cells, which are reported to represent 2% of the stromal vascular fraction (SVF). The infrapatellar fat pad (IFP) is a unique source of tissue, from which human adipose-derived stem cells (hADSCs) have been shown to harbour high chondrogenic potential. This review aims to calculate, based on the literature, the culture time needed before an average knee articular cartilage defect can be treated using stem cells obtained from arthroscopically or openly harvested IFP. Firstly, a systematic literature review was performed to search for studies that included the number of stem cells isolated from the IFP. Subsequent analysis was conducted to identify the amount of IFP tissue harvestable, stem cell count and the overall yield based on the harvesting method. We then determined the minimum time required before treating an average-sized knee articular cartilage defect with IFP-derived hADSCs by using our newly devised equation. The amount of fat tissue, the SVF cell count and the stem cell yield are all lower in arthroscopically harvested IFP tissue compared to that collected using arthrotomy. As an extrapolation, we show that an average knee defect can be treated in 20 or 17 days using arthroscopically or openly harvested IFP-derived hADSCs, respectively. In summary, the systematic review conducted in this study reveals that there is a higher amount of fat tissue, SVF cell count and overall yield (cells/volume or cells/gram) associated with open (arthrotomy) compared to arthroscopic IFP harvest. In addition to these review findings, we demonstrate that our novel framework can give an indication about the culture time needed to scale up IFP-derived stem cells for the treatment of articular cartilage defects based on harvesting method.

## 1. Introduction

Human adipose-derived mesenchymal stem cells (hADSCs) [1,2,3,4,5] are a significant player in tissue engineering, particularly for forming chondrogenic tissue [6,7]. The two primary adipose sources harvested are the subcutaneous fat tissue and the infrapatellar fat pad (IFP). The IFP, which is also known as Hoffa’s fat pad, is a distinct bulk of fat that sits behind the patellar tendon extending into the anterior knee joint [8]. The tissue is functionally structural adipose with little or no metabolic response; furthermore, its contribution to knee function is unclear if any [8]. Stem cells isolated from the IFP have been shown to produce higher chondrogenic potential in comparison to those sourced from subcutaneous fat [9] and possess significant multi-differentiation potential [10]. IFP-derived hADSCs have been used for intra-articular injections into osteoarthritic human knees [11] and for engineered tissue implants for in vivo cartilage repair [12,13].

In the literature, in vivo animal models of osteochondral repair using IFP-derived hADSCs paired with tissue engineering techniques utilise the following treatment process: (1) harvest and isolation of hADSCs, (2) culture expansion of hADSCs, (3) reimplantation of hADSCs combined with a scaffold [12]. A significant concern in these studies has been the use of excessive cell-expansion timeframes (step 2 of the treatment process), which can be performed for many months and passages. Long-term cell expansion in a laboratory is associated with an increased risk of tumorigenic transformation, contamination and exposure to animal-based serum products [14].

When envisioning future clinical translation of regenerative therapies in different individuals, an efficient expansion timeframe needs to be identified. Another key advantage of using an efficient duration for reimplantation is to identify the patient waiting time between surgical procedures (harvest and reimplantation).

To identify the minimum time required for cellular expansion before treatment, three critical pieces of data are needed; (1) the number of hADSCs required for therapy, (2) the IFP-derived hADSCs doubling time, and (3) the number of hADSCs that can be isolated from the IFP.

The cell concentration in healthy cartilage and the defect volume in question dictate how many stem cells are required for therapy. The average number of cells per mL of articular cartilage was described as 1.0 × 10^7^ by Hunziker et al. (2002) [15]. The average knee defect volume in humans is approximately 550 μL (mm^3^) as shown in a hallmark study reporting the findings of diagnostic arthroscopies [16]. Therefore, in an average-sized defect, 5.5 × 10^6^ cells are required for regenerative therapy. The doubling time of IFP-derived hADSCs has been described as roughly 5 days by Garcia et al. (2016) [17]. The remaining piece of data needed is the number of stem cells that can be isolated from the IFP, which has been poorly studied, with no extensive case studies evaluating the cellular yield. When the IFP is digested, the initial cellular population retrieved is a stromal vascular fraction (SVF), which contains many different types of cells. The proportion of SVF cells that are hADSCs has been described in the literature as roughly 2% [18,19], and to specifically isolate these cells, selective plastic adherence and cellular expansion are required as per the International Society for Cellular Therapy [20,21].

A key factor influencing the cellular yield is the amount of fat tissue harvestable, which is limited when using the IFP. The IFP is generally removed along with surrounding connective and fibrous tissue, meaning the overall fat content is less than the amount of total tissue collected. Traditionally the two methods to harvest IFP tissue are keyhole surgery (arthroscopy) or formal opening of the knee joint (open arthrotomy) during arthroplasty. The amount of IPF tissue that can be harvested by either technique is different, given one procedure is minimally invasive and the other is open.

Hence, the overall aim of this systematic review is to identify the culture time needed to scale up IFP-derived stem cells for cartilage regeneration. The data gap within the literature is the evaluation of how many cells there are in the IFP. By identifying this information, we can couple it with the number of cells needed for therapy and the doubling rate (described above) to ascertain the minimum timeframe required for cellular expansion before reimplantation and therapy. This is calculated using a modified derivation of the cellular doubling time equation [17] and is detailed in the methods section.

The specific outcomes that will be assessed are; the amount of fat tissue retrievable, the cellular yield and finally, the number of SVF cells obtainable. These outcomes will be evaluated by the method of harvest used (arthroscopic or open arthrotomy).

Finally, using the sourced data from the systematic review, the minimum time required for cellular expansion prior to treating an average-sized knee defect is determined using a newly derived equation by the authors.

## 2. Materials and Methods

### 2.1. Study Design and Literature Search

A systematic literature review based on the Preferred Reporting Items for Systematic Reviews and Meta-Analyses (PRISMA) guidelines [22] was conducted on the 15th of October, 2019.

Two independent investigators (SF, AY) searched electronic databases (PubMed, Cochrane Central Register of Controlled Trials (CENTRAL) and Embase) with English language restrictions. 

The following keywords were used: “infrapatellar fat pad” AND “mesenchymal stem cell” OR “adipose-derived stem cell”. Duplicates were removed and reference lists were also searched manually for additional studies. Our search combined the following search term groups;

1. Infrapatellar fat pad
2. Stem cell OR mesenchymal stem cell OR adipose-derived mesenchymal stem cell

Using the Boolean operator OR within the search group and then combining the two groups with the operator AND we formulated our search design.

### 2.2. Study Selection and Eligibility Criteria

The search was restricted to human studies written in English published within the last 20 years (1998 onwards). Search results from each database were then imported into EndNote bibliographic management software (Thomson Reuters, Endnote version X7, Ottawa, ON, Canada) and duplicates were then identified. Level one screening (SF and AY) was performed by looking at article titles and abstract records and screened based on carefully selected inclusion and exclusion criteria, as shown in Table 1.

The remaining pool of articles underwent level 2 screening (same exclusion parameters as level 1). The full text of these articles was screened by two independent reviewers (SF and AY) and relevant articles selected.

Differences in opinion were resolved by consensus. Additional hand searching of reference lists of included full-text articles were identified and added for inclusion if appropriate.

### 2.3. Data Extraction

Two independent reviewers (SF, AY) obtained relevant data and assessed the accuracy of the screened articles. In the event that both authors could not reach an agreement, a third author (PC) decided. The following information was extracted from each study: first author’s name, year of publication, country, study design, participant eligibility, patient demographics, study sample size, harvest procedure, surgical indication, the quantity of IFP tissue and cells yielded per study. Contact with corresponding authors was also attempted when deemed necessary to verify the accuracy of the data and obtain further data for the analysis.

### 2.4. Quality Control and Assessment

Quality control procedures for articles included level 1 (titles/abstracts) and level 2 (full text) screening for eligibility according to inclusion and exclusion criteria. Level 2 screening, as mentioned prior, was performed independently by two researchers. Articles for which there was any uncertainty about inclusion were discussed with a third researcher. Data were extracted from full-text versions of articles.

### 2.5. Minimum Time Required for Regenerative Therapy Using IFP-Derived hADSCs

This is calculated using the data sourced from the systematic review and the following equation, which is a derivation from the cell population doubling time equation [17]. The number of days in culture (*t*2−*t*1) from the original equation corresponds to the minimum time (days) used in this formula.

Minimum time (days) = [DT × ln(*n*2/*n*1)]/[ln (2)] (1)

**Theorem** **1.** *Where DT refers to the cell doubling time, n2 is the total number of hADSCs required for therapy and n1 is the number of hADSCs initially isolated. The number of hADSCs is calculated as 2% of the SVF population, based on literature described in the introduction.*

## 3. Results

### 3.1. Search Results

**Figure 1:**
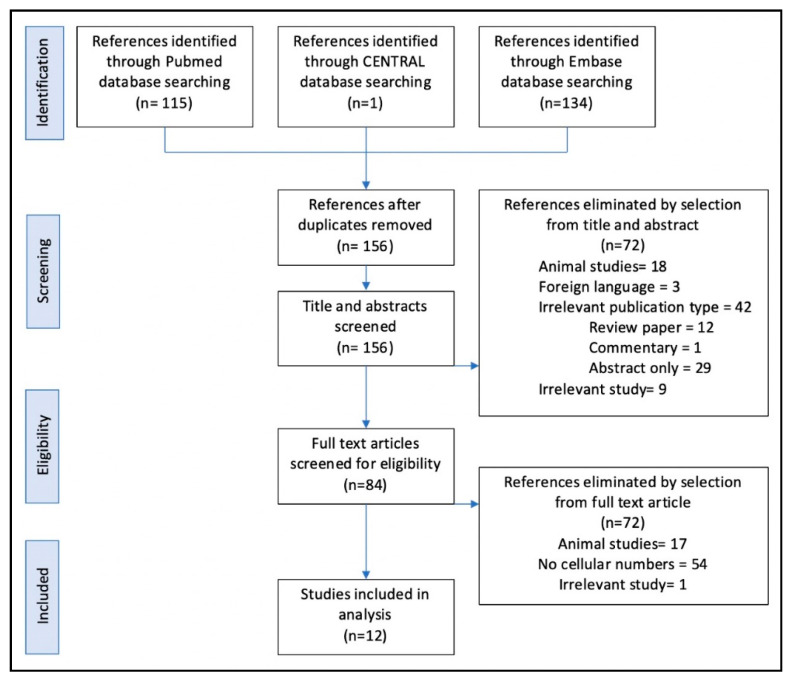
Article screening process, conducted using the PRISMA guidelines, Liberati et al., BMJ, 2009 [22].

Using the search strategy described above, 156 articles were obtained, of which 72 were screened at level 1 after review of title and abstracts. Of the remaining 84 articles, after reviewing the full text, a further 72 articles were screened out at level 2. References were checked in the remaining 12 articles, and no new articles were added to the final list. Reasons for article exclusions are detailed in Figure 1.

### 3.2. Included Studies

All 12 included articles were controlled single-institution studies (Table 2). Of the 12 isolated studies, 11 were retrospective [23,24,25,26,27,28,29,30,31,32,33] and one was a prospective cohort study [34]. There was a total of 228 patient IFPs assessed in the 12 studies. The patient ages ranged between 17 and 89. From the six studies indicating gender [23,24,25,27,31,34], 77 (62%) patients were female and 48 (38%) were male.

The amount of IFP sourced was described by volume in three (25%) studies [23,25,28], by weight in six (50%) studies [26,30,31,32,33,34] and not described in three (25%) studies [24,27,29]. The range of IFP harvested by volume was described between 2.5 and 25 mL, while the range, when described by weight, was between 5 and 26.3 g. The number of cells (SVF) at isolation was reported in 10 (92%) studies [23,24,25,26,27,28,29,30,31,32,34] and was described as either the total number of cells isolated, cells/g or cells/fat pad. The remaining study [33] did not report the number of cells at isolation; instead, the number of cells obtained after two passages of tissue culture was described. The yield of cells will be presented more in detail below when specifically assessed based on harvesting method.

### 3.3. Study Outcomes

#### 3.3.1. Arthroscopic Based Harvest of IFP 

There were 3 (25%) studies [25,27,34] that investigated arthroscopically harvested IFP. In total, 53 patient IFPs were assessed in these three studies. Patient ages ranged between 17 and 69. All three papers indicated gender, with 27 (51%) patients being female and 26 (49%) being male. The amount of IFP sourced was described by volume in one (33%) study [25], by weight in 1 (33%) study [34] and not specified in the remaining (33%) study [27]. One study presented the cellular count as a mesenchymal stem cell (MSC) count; however, this conflicted with their methodology and was likely to represent the SVF count [34].

The study [25] describing the volume of tissue harvested assessed 3 mL of IFP, from which an average of 7.0 × 10^5^ (6.0–8.0) SVF cells were retrieved. The yield by volume in this study was, on average, 2.3 × 10^5^ cells/mL of IFP tissue. The study describing the weight of IFP tissue harvested averaged 9.4 g (6.9–11.2), with an average SVF count of 1.89 × 10^6^ (1.2–2.3 × 10^6^). The yield by weight in these studies was, on average, 2.01 × 10^5^ cells/g of IFP.

The overall yield in all these studies was calculated to be 1.2 × 10^6^ cells per IFP (0.93–2.3 × 10^6^), irrespective of whether volume or weight was used to describe the harvested amount.

#### 3.3.2. Open Arthrotomy Based Harvest of IFP

There were 10 (77%) studies [23,24,25,26,29,30,31] that investigated the open harvest of IFP using arthrotomy. A total of 175 patient IFPs were assessed, with ages ranging between 37 and 89. Gender was indicated in four studies [23,24,25,31]; 40 (65%) patients were female and 22 (35%) were male. The amount of IFP sourced was described by volume in three (30%) studies [23,25,28], by weight in five (50%) studies [26,30,31,32,33] and not described in two (20%) studies. One study did not describe the SVF yield obtained during cellular isolation.

The volume of tissue harvested was on average 4.4 mL of IFP (2.5–25); in these studies, the number of SVF cells isolated on average was 7.9 × 10^5^ cells (0.1–120). The yield by volume in these studies on average was 1.8 × 10^5^ cells/mL of IFP tissue. The weight of IFP tissue harvested was on average 15.2 g (5.0–26.3); in these studies, the number of SVF cells isolated on average was 4.5 × 10^6^ cells (3.94–5.5). The yield by weight in these studies was, on average, 3.0 × 10^5^ cells/g of IFP.

The overall yield in all these studies was calculated to be 3.0 × 10^6^ cells per IFP (2.8–4.0 × 10^6^), irrespective of whether volume or weight was used to describe the harvested amount.

## 4. Discussion

Sourced papers from the systematic review originated from Europe, Asia and America, representing a diverse pool of data; furthermore, a good spread between gender and age groups was obtained. The difference in surgical procedures amongst different regions is an important consideration, although for IFP harvest, arthroscopic and arthrotomy related techniques are generally the same worldwide. Primary osteoarthritis and ligament damage were the indications for treatment in these studies and given that IFP is opportunistically harvested, the lack of literature/data on the IFP of healthy individuals remains a key limitation in this field.

The volume of tissue harvested by arthroscopy was, on average, 3 mL compared to 4.4 mL of IFP using open arthrotomy. The weight of tissue harvested was on average, 9.3 g compared to 15.2 g of IFP tissue using arthroscopy and arthrotomy, respectively. Although safe and efficiently performed, the restricted access and exposure associated with arthroscopic surgery appear to significantly limit the amount of tissue harvestable as compared to arthrotomy, where the whole IFP can be removed easily.

The overall cellular yield was also lower in tissue harvested from arthroscopy compared to arthrotomy, irrespective of whether the amount was described by volume or weight. The overall yield of SVF cells was, on average, 1.2 × 10^6^ and 3.0 × 10^6^ cells per IFP when using arthroscopy and an open arthrotomy, respectively. Arthroscopically harvested tissue contains a fluidic component that is specific to the technique, as opposed to harvest via open arthrotomy, in which the entire IFP is retrieved en mass without the mixing or introduction of any fluid. This dilution does not affect the total number of cells retrieved; however, it can lead to a lower overall yield per unit of tissue. Another consideration as to why the yield is lower arthroscopically is whether the roughness involved with arthroscopic drainage through a tube may lead to cellular damage and loss, therefore, reducing the overall live cell count and yield.

Analysing all the IFP samples, on average 1.2 × 10^6^ and 3.0 × 10^6^ SVF cells were isolated using arthroscopic and open harvest respectively. Therefore, using the formula described in the methods, the earliest time point in which an average-sized knee defect can be treated using hADSCs sourced from arthroscopic or open IFP harvest was calculated to be 40 (35–41 days) and 33 days (31–33 days), respectively (calculation shown in Supplementary Section S1). If both IFPs (one in each human knee) are utilised, treatment can be performed in 20 (17–21 days) and 17 (16–17 days) days respectively. A limitation to the use of this calculation is the high variability seen in SVF counts, the use of an assumed doubling time and assumed hADSCs percentage within the SVF. In culmination these differences can add up to make a large spread of results, to control this, in our calculation, we used the standard deviation range of the SVF yields to present a range of days that it could take to scale cells up. Ultimately, this equation can be used as a guideline and the use of more defined input data allows for a more precise end calculation.

Although it takes longer culturing time to scale up arthroscopically harvested IFP-derived hADSCs to treat articular cartilage defects, this approach is the most likely to be adopted when envisioning future clinical translation of regenerative therapies for osteochondral repair. This is due to the minimally invasive nature of the technique, which is associated with less surgical risk compared to an open arthrotomy and enables the development of a same-day surgical repair option.

Using the same calculation described in this review, the culture time required to scale IFP-derived stem cells prior to cartilage repair can be determined for any defect volume. Therefore, this work represents a framework that has never been described in the literature. The next step is to further validate this framework by evaluating the cells obtained after specific expansion times with respect to phenotype and function. This can be performed by utilizing an in vitro study to prove that cells expanded for specific timeframes display the hADSCs phenotype and can then be successfully driven into chondrogenesis. If this can be achieved in vitro, a subsequent in vivo model could be performed to ultimately prove chondrogenic regeneration using these adapted time calculations in the native joint environment.

## 5. Conclusions

This systematic review shows that there is a higher amount of fat tissue, SVF cell count and overall yield (cells/volume or cells/gram) associated with open (arthrotomy) compared to arthroscopic IFP harvest. As an extrapolation, it takes an average of 20 or 17 days to scale up arthroscopically or openly harvested IFP-derived hADSCs, respectively, to reach a sufficient amount of cells enabling cartilage defect repair.

## Figures and Tables

**Table 1:** Inclusion and exclusion criteria.

Inclusion Criteria	Exclusion Criteria
Human participants ≥ 18 years old	Conference abstracts and non-original papers (commentaries, reviews, letters or editorials)
Studies written in English	Animal studies
Clinical studies, multi-centre studies, observational studies, clinical trials, randomised control trials, meta-analyses and systematic reviews	Studies conducted in children, adolescents or pregnant women
Measurement of MSCs derived from IFP only	Non-IFP-derived MSCs or studies lacking specific data relating to IFP or MSC numbers
Explicit measurement of the quantitative value of MSCs found per participant	Off-topic studies

**Table 2:** Overview of each study included in the review. TKA—Total knee arthroplasty, OA—Osteoarthritis, SVF—Stromal vascular fraction, IFP—Infrapatellar fat pad. Data is represented as the mean +/− standard deviation (SD).

Study	Country	Patient Demographics	Harvest Procedure (Surgical Indication)	IFP Tissue Yield	Cellular Data/Yield
*Mantripragada* et al. *2019* [23]	USA	n = 28 Mean age: 63.1 (37–82) F = 16, M = 12	TKA (Idiopathic OA)	2.5 mL of IFP	SVF = 4320 cells per mL of IFP
*Lopez-Ruiz* et al. *2018* [24]	Spain	n = 8 Mean age: 66.9 (57–74) F = 4, M = 4	TKA (Idiopathic OA)	Not described	SVF = 1.1 × 10^5^ cells per gram of IFP (0.9–1.3 × 10^5^)
*Bravo* et al. *2019* [25]	Spain	n = 21 ≥ Arthroscopic ACL repair Median age: 32 (21–50) F = 4, M = 17 n = 21 ≥ TKA Median age: 74 (57–84) F = 15, M = 6	Arthroscopic repair (ACL rupture) Or TKA (OA)	≥3 mL of adipose extracted from IFP ≥3 mL of adipose extracted from IFP	6.0 × 10^5^–8.0 × 10^5^ cells
*Wang* et al. *2017* [26]	China	n = 4	TKA (No stated indication)	10 g of IFP	Primary culture (SVF) ≥ Approx. 5 × 10^5^/g of IFP
*Dragoo* et al. *2017* [27]	USA	n = 7 Median age: 35.14 (17–52) F = 6, M = 1	Arthroscopic repair (ACL rupture with no evidence of OA)	Not described	SVF = 4.86 × 10^5^ cells 0.19–0.33 × 10^5^ cells per gram of IFP
*Munoz-Criado* et al. *2017* [29]	Spain	n = 24 Ages: 50–80	TKA (Idiopathic OA)	Not described	SVF = 7.8 × 10^5^ ± 2.8 × 10^5^ cells
*Neri* et al. *2017* [30]	Italy	n = 11 Mean age: 69.4 ± 6.5	TKA (Idiopathic OA)	5–15 g IFP obtained	SVF = Average of 7.0 × 10^5^ cells per gram of IFP
*Tangchitphisut* et al. *2016* [31]	Thailand	n = 5 Mean age: 65.8 (53–77) F = 5, M = 0	TKA (Idiopathic OA)	Average IFP weight 12.12 ± 2.57 g (8.48–14.75)	SVF: 3.94 × 10^6^ ± 3.73 × 10^5^ cells
*Koh* et al. *2012* [34]	South Korea	n = 25 Mean age: 54.2 ± 9.3 (34–69) F = 17, M = 8	Arthroscopic harvest (secondary OA)	Average IFP weight 9.4 g (6.9–11.2)	SVF (described as MSC count in the paper) = 1.89 × 10^6^ (1.2–2.3 × 10^6^)
*Jurgens* et al. *2009* [32]	Netherlands	n = 53 Median age: 72 (43–89)	TKA (Idiopathic OA)	Average IFP weight 15.1 ± 5.8 g (8.7–26.3)	SVF: 4.0 × 10^6^ ± 4.45 × 10^5^ cells
*Dragoo* et al. *2003* [28]	USA	n = 5 Mean age: 74 (53 to 86)	TKA (no indication stated)	Average IFP volume 20.6 mL (15–25)	SVF mean yield of 5.5 × 10^6^ extracted cells per IFP (2.0 × 10^6^ to 1.2 × 10^7^)
*Wickham* et al. *2003* [33]	USA	n = 16 Mean age: 68 ± 11.1 (49–82)	TKA (no indication stated)	Average IFP weight 21.5 ± 8.8 g	Not described

## Supplementary Materials

The following are available online at https://www.mdpi.com/2306-5354/7/3/69/s1, Section S1: calculation of the minimum time required for regenerative therapy using IFP-derived hADSCs.

## Abbreviations

SVF	Stromal vascular fraction
IFP	Infrapatellar fat pad
MSCs	Mesenchymal stem cells
hADSCs	Human adipose-derived mesenchymal stem cell
CENTRAL	Cochrane Central Register of Controlled Trials
PRISMA	Preferred Reporting Items for Systematic Reviews and Meta-Analyses
