# Supplementary material for: Culture Time Needed to Scale up Infrapatellar Fat Pad Derived Stem Cells for Cartilage Regeneration: A Systematic Review
Source: Bioengineering (Basel). 2020 Jul 4;7(3):69. doi: 10.3390/bioengineering7030069 (PMC7552776; doi:10.3390/bioengineering7030069)
Supplement: Supplementary file 1 [file bioengineering-07-00069-s001.pdf]

bioengineering

MDPI

Supplementary Section:

S1: Calculation of the Minimum Time Required for Regenerative Therapy using IFP-derived hADSCs

Doubling time (DT) = For IFP-derived hADSCs roughly 5 days

Number of hADSCs required for therapy (n2) = Average defect size (550 uL) × normal concentration of chondrocytes (1.0×10^7 cells/1 mL)

$= 550 \times 1.0 \times 10^7/1000$

$= 5.5 \times 10^6$ cells

Number of hADSCs initially isolated (n1) = The average number of SVF cells per IFP identified in the systematic review was used, and 2% of the SVF population is used to account for hADSCs.

n1 (arthroscopy) = $0.02 \times 1.2 \times 10^6$ ($0.93 - 2.3 \times 10^6$), = 2.4×10^4 stem cells [$1.86 - 4.6 \times 10^4$]

n1 (arthrotomy) = $0.02 \times 3.0 \times 10^6$ ($2.8 - 4.0 \times 10^6$), = 6.0×10^4 stem cells [$5.6 - 8.0 \times 10^4$]

Therefore, minimum time required for cells isolated using arthroscopy:

Minimum time (days) = [DT x ln($n2$/$n1$)]/[ln (2)]

= [5 x ln ($5.5 \times 10^6/2.4 \times 10^4$)]/[ln (2)]

= 39.2 [34.5–41.0]**Rounded up to 40 days [35–41 days] using one IFP, 20 days* [17–21 days] *using two IFPs (humans have two IFPs)* [10^4]

Therefore, minimum time required for cells isolated using arthrotomy:

Minimum time (days) = [DT × ln($n2$/$n1$)]/[ln (2)]

= [5 × ln (5.5 x $10^6/6.0 \times 10^4$)]/[ln (2)]

= 32.60 [30.5–33.1] **Rounded up to 33 days [31–33 days] using one IFP, 17 days [16–17 days] using two IFPs*

** Error margins are represented by +/– the Standard deviation (SD).*
